# Treatment in Borderline Class III Malocclusion: Orthodontic Camouflage (Extraction) Versus Orthognathic Surgery 

**DOI:** 10.2174/1874210600802010038

**Published:** 2008-03-08

**Authors:** A-Bakr M. Rabie, Ricky W.K. Wong, G.U. Min

**Affiliations:** 1 Discipline of Orthodontics, Faculty of Dentistry, The University of Hong Kong, 2/F Prince Philip Dental Hospital, 34 Hospital Road, Sai Ying Pun, HKSAR, China; 2 Discipline of Orthodontics, Faculty of Dentistry, The University of Hong Kong, 2/F Prince Philip Dental Hospital, 34 Hospital Road, Sai Ying Pun, HKSAR, China; 3 Department of Stomatology, the Second People Hospital, Shenzhen, China

**Keywords:** Class III malocclusion, camouflage, orthognathic surgery

## Abstract

**Aims:**

To investigate the differences in morphological characteristics of borderline class III patients who had undergone camouflage orthodontic treatment or orthognathic surgery, and to compare the treatment effects between these two modalities.

**Materials and Methods:**

Cephalograms of 25 patients (13 orthodontic, 12 surgical) with class III malocclusion were analyzed. All had a pretreatment ANB angle greater than -5º.

**Results:**

Using discriminant analysis, only Holdaway angle was selected to differentiate patients in the pretreatment stage. Seventy-two per cent patients were correctly classified. In the orthodontic group, reverse overjet was corrected by retraction of the lower incisors and downward and backward rotation of the mandible. The surgical group was corrected by setback of the lower anterior dentoalveolus and uprighting of the lower incisors. No difference was found in posttreatment soft tissue measurements between the two groups.

**Conclusions:**

Twelve degree for the Holdaway angle can be a guideline in determining the treatment modalities for borderline class III patients, but the preferences of operators and patients are also important. (2) Both therapeutic options should highlight changes in the lower dentoalveolus and lower incisors. (3) Both treatment modalities can achieve satisfactory improvements to the people.

## INTRODUCTION

Class III malocclusion is far more prevalent in Asian countries than in the West. (Graber Mosby 2005) [1]. The incidence of anterior crossbite is 2.3-13 per cent among Japanese, 9.4-19 per cent among Koreans and 12.8 per cent among Chinese (Fu ZHKQYXZZ 2002) [2] (and 14.5 per cent in southern Chinese) (Chan AJODO 1974) [3]. Accordingly, class III malocclusions account for a large proportion of orthodontic patients in these countries—for example, 33 per cent of orthodontic patients in Japan and 20 per cent in China. (Fu ZHKQYXZZ 2002) [2] In contrast, the prevalence of class III malocclusion in the United States is only about 1.0 per cent of the total population, and only 5 per cent of orthodontic patients. (Graber Mosby 2005) [1].

There are three main treatment options for skeletal class III malocclusion: growth modification, dentoalveolar compensation (orthodontic camouflage), and orthognathic surgery. Growth modification should be commenced before the pubertal growth spurt, after this spurt, only the latter two options are possible. In such cases, however, how should clinicians determine whether or not patients are suitable for surgery?

Kerr *et al*(Kerr BJO 1992) [4] tried to establish some cephalometric yardsticks in adult patients with class III malocclusion to find objective criteria for treatment options. These researchers suggested that surgery should be performed for patients with an ANB angle of less than -4°, a maxillary/mandibular (M/M) ratio of 0.84, an inclination of the lower incisors to the mandibular of 83°, and a Holdaway angle of 3.5°. In 2002, a formula was developed to determine whether patients with class III malocclusion underwent either orthodontic treatment or orthognathic surgery, on the basis on the four variables: Wits appraisal, length of the anterior cranial base, M/M ratio, and lower gonial angle. (Stellzig-Eisenhauer AJODO 2002) [5]. However, these two studies did not provide methods to specifically distinguish between patients with borderline surgical-orthodontic class III malocclusion. Furthermore, Cassidy *et al*(Cassidy AJODO 1993) [6] investigated borderline class II division 1 malocclusions and found that characteristics on which the orthodontic or surgical decision had been based were similar for 27 adult orthodontic and 26 adult surgical patients. The treatment choices largely depended on the clinicians preferences.

Therefore, it is essential to evaluate borderline class III patients very carefully. The objectives of this study were to investigate the different morphological characteristics of borderline surgical-orthodontic class III patients and to compare treatment outcomes between the 2 patient groups. Data from this study will help clinicians in treatment planning.

## MATERIAL AND METHODS

### Orthodontic Group Samples 

In this retrospective study, we investigated the treatment records from patients who attended the postgraduate clinic of the Discipline of Paediatric Dentistry and Orthodontics, Faculty of Dentistry, The University of Hong Kong, between 2003 and 2006. All anterior crossbite patients who had been treated by orthodontic means alone were included for selection. The selection criteria were as follows:

1. Southern Chinese
2. Orthodontic treatment performed
3. No obvious transversal discrepancy, non-cleft
4. ANB< 1^°^ or Wits appraisal < -7.5 mm, as checked from pretreatment cephalometric records. These limits of the ANB angle and Wits appraisal for skeletal class III malocclusion were derived from cephalometric norms of southern Chinese. (Cooke EJO 1988) [7].

**Fig. (1):**
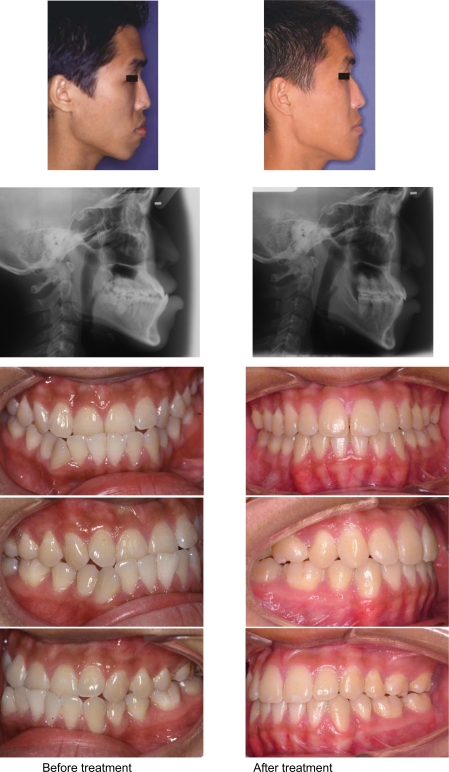
Extra-oral, intra-oral and cephalograms of one orthodontic sample before and after treatment

Twenty patients (13 extraction, 7 non-extraction) satisfied the inclusion criteria, but because the mechanisms of extraction and non-extraction treatment were different, and the sample for non-extraction was small, the non-extraction cases were excluded. Therefore, 13 patients (8 males and 5 females; mean age, 16.2±4.9 years) who underwent extraction were selected as the orthodontic group (Fig. **1**). The details of the extraction protocols are shown in Table **1**. Since all of the pretreatment ANB angles of these patients were greater -5°, this angle was used as the criterion for the surgical sample.

### Surgical Group Samples

**Fig. (2):**
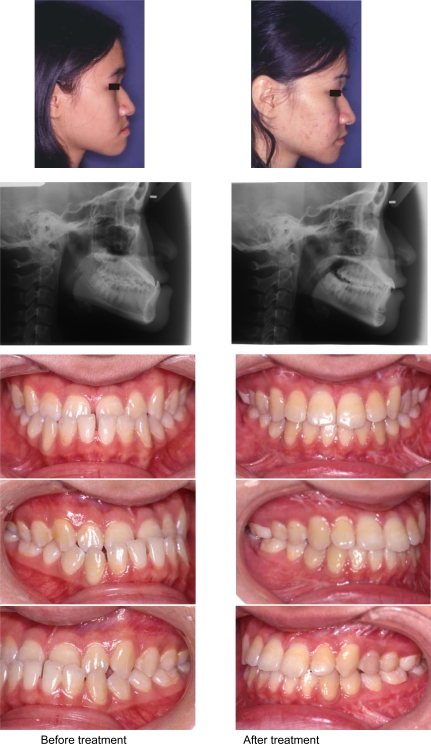
Extra-oral, intra-oral and cephalograms of one surgical sample before and after treatment

Patients with anterior crossbite who attended the postgraduate clinic of the Discipline of Oral and Maxillofacial Surgery, Faculty of Dentistry, The University of Hong Kong, between 2002 and 2006 for surgical treatment were included for selection. The reason for including one more year than the orthodontic group was to obtain a comparable sample size. The selection criteria were the same as those of the orthodontic group except for an ANB angle of greater than -5º. Twelve patients (2 males and 10 females; mean age, 19.4±4.9 years) were included in the surgical sample (Fig. **2**). In them, nine patients had undergone bimaxillary surgery, two patients had undergone mandibular surgery only, and the rest one had undergone maxillary surgery only.

### Cephalometric Analyses

All lateral cephalograms that had been obtained before and after treatment were scanned (Epson Expression 1649-XL; Seiko Epson Corp., Japan), traced, and digitized by one investigator. A commercial cephalometric program (Winceph 7.0; Rise Corp., Japan) was used to study the cephalometric landmarks shown in Fig. (**3**). Twenty-four angular, one linear, and three proportional measures were used in this study, most of which were the same as those used in two previous studies, (Kerr BJO 1992) [4] (Stellzig-Eisenhauer AJODO 2002) [5] except for measurements of the NPog-SN angle, Go-Me/S-N ratio, and Z angle.

Because there is an obvious sexual dimorphism among class III patients (Ngan IJAOOS 1997) [8] (Baccetti AO 2005) [9] and this study combined males and females, only angles, proportional measurements, and Wits appraisal were measured in this investigation. All these variables have previously been proven to be independent of sex.

### Method Error

Cephalograms from 10 randomly chosen patients were retraced and redigitized on two different occasions separated by a 2-week interval. The method error was calculated using Dahlberg’s formula (Houston AJODO 1983) [10]:

$$\mathrm{ME}=\sqrt{\sum d^{2}/2n}$$

where *d* is the difference between 2 registrations of a pair, and *n* is the number of double registrations. The random errors ranged from 0.46° to 1.79° for angular variables, from 0.02 to 1.68 for ratio variables, and 1.66 mm for Wits appraisal measurements.

### Statistical Analyses

Mann-Whitney U test was applied to compare variables between the orthodontic and surgical groups. Wilcoxon signed rank test was used to compare pretreatment and posttreatment variables for each group. Stepwise discriminant analysis was applied to identify the possible variables that best separated the pretreatment groups. The data were analyzed by using SPSS for Windows, version 13.0 (SPSS Inc., Chicago, Ill). Cutoffs for statistical significance were taken as P<0.05,<0.01, and <0.001.

## RESULTS

### Comparison of Pretreatment Values between Orthodontic and Surgical Group

Table **2** shows that significant differences (P<0.05) were found in three measurements: the Go-Me/S-N ratio, U1-L1 angle, and Holdaway angle. Stepwise discriminant analysis identified only one variable that distinguished between patients suitable for orthodontics from those suitable for surgery. That factor was the Holdaway angle (F likelihood to remove = .014). On the basis of the unstandardized discriminant function coefficients of the selected variable, along with a calculated constant, the following equation for individual scores was developed:

Individual score = -2.989+0.24×(Holdaway angle)

The critical score was 12°, which was the mean centroid of the two groups. This implies that a new borderline class III malocclusion patient with a Holdaway angle greater than 12° would be treated successfully by orthodontics alone. On the contrary, a new patient with a Holdaway angle of less than 12° should be treated by combined surgical-orthodontic treatment. In this way, 72 per cent of the patients were correctly classified. Three patients of the orthodontic group and four of the surgical group had been misclassified (Table **3**).

### Comparison of Pretreatment and Posttreatment Values within Orthodontic Group

Significant increases were found in measurements for the PoOr-NBa angle (P<0.05), Go-Me/S-N ratio (P<0.01), lower facial height proportion (P<0.05), interincisal angle (P<0.01), and Z angle (P<0.01). The decreases in the gonion angle (P<0.01), upper gonion angle (P<0.01), and L1-ML angle (P<0.01) were also statistically significant (Table **4**). After tracings were superimposed along the anterior cranial base at the nasion, the posttreatment tracing showed a more prognathic mandible, increased lower facial height, retracted lower incisors, and retruded lower lip than the pretreatment tracing.

**Fig. (6):**
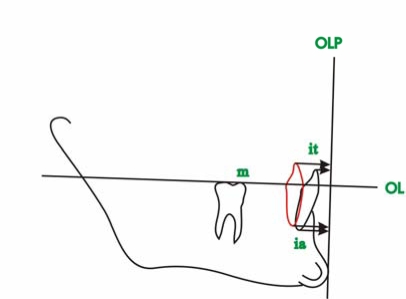
Lower incisor position change. Mandibular tracings super-imposed on anterior contour, internal cortical surface of the sym-physis and mandibular canal. Pretreatment (black line), posttreat-ment (red line) m   The mesiobuccal cusp tip of the mandibular permanent first molar it   The incisal tip of the most prominent mandibular central incisor ii   The incisal apex of the most prominent mandibular central incisor OL   Occlusal line, a line through m and the buccal cusp tip of the mandibular first premolar OLP   Occlusal line perpendiculare, a line perpendicular to OL through the most anterior point of the bony chin symphysis

To assess the movement pattern of the lower incisors, an analysis based on sagittal-occlusion analysis (Pancherz AJODO 1985) [11] and a ‘Pitchfork diagram’ (Johnston BJO 1996) [12] was conducted (Fig. **6**). Table **5** shows that lower incisors were retracted 4.9 mm in the incisal tip and 1.9 mm in the incisal apex.

### Comparison of Pretreatment and Posttreatment Values within Surgical Group

After surgical treatment, samples in this group showed significant differences in the following measures: decreased SNB angle (P<0.01), NPog-SN angle (P<0.05); increased L1-ML angle, and Holdaway angle (P<0.01); highly increased ANB angle (P<0.01); and Wits appraisal and M/M ratio (P<0.01) (Table **6**).

Fig. (**7**) shows the changes after surgery, mainly the setback of the mandibular dentoalveolus and chin, and the uprighting and retraction of the lower lip.

### Comparison of Posttreatment Values between Orthodontic Group and Surgical Group

The posttreatment comparison of the two groups and superimposition of averaged tracings are shown in Table **7** and Fig. (**8**), respectively. Significant differences were found in the ANB angle (P<0.01), M/M ratio (P<0.05), NAPog angle (P<0.01), L1-ML angle (P<0.05), and U1-L1 angle (P<0.01). Apart from the changes with respect to the hard tissues mentioned above, there were no significant differences in the two soft tissue measurements. Hence, both lateral profile improvements were esthetically harmonic, although the lower lip was more distally positioned bodily in the surgical group than in the orthodontic group.

## DISCUSSION

Class III malocclusion is among the most difficult de-formities to be corrected, especially using orthodontic means alone. This study focused on successfully treated borderline class III patients to provide some treatment guidelines that can help in treatment decisions for this malocclusion.

Borderline surgical/orthodontic cases refer to patients with mild to moderate skeletal problems that can be treated by either orthodontic or surgical means. Cassidy (Cassidy AJODO 1993) [6]defined “borderline cases” as those patients who were similar with respect to the characteristics on which the orthodontic/surgical decision appeared to have been based. In this study, the common characteristic of both groups was the same ANB angle range (above -5º).

Although many studies (Jacobson AO 1988) [13] suggested combining the ANB angle and Wits appraisal to evaluate the sagittal discrepancy, the ANB angle is still a simpler and more commonly used variable. In this study, the values of Wits appraisal showed no significant difference between the two groups before treatment. This finding indicated that the sagittal discrepancy was actually in the same range in the two groups.

All of the pretreatment cephalograms used in this study were taken in the CO position, regardless of whether or not the patients had mandibular anterior displacement. Gravely (Gravely BJO 1984) [14] found that a conventional cephalograph taken in the CO position could reasonably reflect the skeletal pattern in most cases. He doubted whether a second cephalograph taken with the incisors held edge to edge provided sufficient additional information.

Even though all the patients were in the same range of sagittal discrepancy, several significant differences could still be found between orthodontic and surgical patients before treatment. A lower Holdaway angle, higher Go-Me:S-N ratio, and increased U1-L1 angle indicated a more prognathic mandible, greater compensation of incisors, and a more concave profile in the surgical group. Discriminant analysis showed that for the measurement of the profile, the Holdaway angle, was the most crucial variable to classify patients. The threshold value was 12º, which meant that if one patient had a Holdaway angle of greater than 12º, he or she would most likely to be successfully treated by orthodontics. This value was much higher than the 3.5º suggested by Kerr. (Kerr BJO 1992) [4] The variable racial composition of the sample probably contributed to this difference.

The proportion of correctly classified patients was 72 per cent—less than the 92 per cent found in Stellzig-Eisenharer’s study. (Stellzig-Eisenhauer AJODO 2002) [5] A possible reason for the lower proportion is that morphology may not be the only factor that determines the treatment decision, especilally in borderline cases. The preference of patients and operators also could affect the final option chosen. Proffit (Proffit IJAOOS 1990) [15] found that psychologic rather than morphologic characteristics probably were the major influence on whether or not an individual decided to accept surgery. Bell (Bell AJODO 1985) [16] also pointed out that the decision of surgery was mainly related to the self-perception of patients. In addition, the preference of operators was also important. Cassidy (Cassidy AJODO 1993) [6] found that in borderline class II patients, the final treatment choice was highly depended on which clinician the patient happened to contact. Bell (Bell AJODO 1985) [16] opined that surgeons and orthodontists may differ in recommendations for surgical correction. Consequently, a Holdaway angle of 12º can be only a rough guideline to help in treatment planning. Nevertheless, the preference of patients should also be considered.

It is commonly believed that successful camouflage treatment for class III malocclusion can be achieved by proclination of maxillary incisors, retrusion of mandibular incisors, and downward and backward rotation of mandible. In this study, as all of the patients were extraction cases the upper incisors showed mild retroclination rather than proclination. This finding was similar to that reported by Battagel. (Battagel EJO 1991) [17] Thus, the retraction of the lower incisors and rotation of the mandible were crucial for crossbite correction. In a detailed analysis of the mode of movement of the lower incisors, the crown tips and root apices of the lower incisors were retracted by 4.9 mm and 1.9 mm, respectively, and this retraction was combined with tipping and bodily movement. The bodily movement of the roots was important in preventing over retroclination of the lower incisors. In order to do that, lingual root torque should be applied to the lower incisors during treatment.

After distalization of the lower incisors, the facial convexity was increased accordingly, which contributed to the significant change in Z angle. Unlike the Holdaway angle, the Z angle (Merrifield AJODO 1966) [18] uses the more protruded lip (upper or lower) rather than the upper lip to establish the profile line, whereas in class III malocclusion, lower lip is always more protruded than upper lip, this is why in this study the change of Z angle showed a statistically significant difference, whereas the Holdaway angle did not.

Surgical correction of class III malocclusion can be achieved by mandibular setback, maxillary advancement, or a combination of both procedures. In this study, 8 of 12 patient underwent bimaxillary surgery. The main skeletal changes after surgery were setback of the mandibular dentoalveolus and the uprighting of the lower incisors, whereas the skeletal base of both jaws did not show any significant changes. The setback of the dental alveolus in the mandibular anterior region contributed to the decreased ANB angle, increased Wits appraisal, and M/M ratio. Although the mandibular length did not show any obvious decrease, the chin point seemed to follow the setback of the apical base of the incisors, as manifested by the decreased NPog-SN angle. Possible reasons accounting for this finding include distal displacement of the mandible and remodeling of the chin point after anterior subapical osteotomy. The improvement in the Holdaway angle demonstrates the consequent adaptation of the soft tissue. The unchanged Z angle, however, may be explained by the decompensation of the lower incisors, which contributes to the stable position of the lower lip related to the chin point.

Because the profile rather than occlusion may be the main focus of concern for class III patients, (Bailey IJAOOS 2001) [19] improvement in the profile should play a major role in the evaluation of treatment outcomes. In this study, both patient groups showed obvious improvements in their profile. Although the lack of significant difference in Holdaway angle and Z angle between the two groups does not mean that orthodontic treatment can achieve the same degree of improvement as the surgical approach, orthodontics can still change the profile to achieve an acceptable esthetic effect. The most pronounced characteristic of soft tissue change in this study was limited to the lower lip rather than both lips, regardless of treatment group. This finding was different from that of previous studies, in which a change in both lips was described. (Lew IJAOOS 1990) [20] The sampling technique of the borderline and extraction cases may account for this difference.

## CONCLUSIONS

In conclusion, the present study indicates that (1) the Holdaway angle can be a reliable guide in determining the treatment modality for patients who represent borderline class III surgical cases; (2) the treatment effect of both treatment options should emphasize a change in the lower jaw and lower incisors; and (3) among correctly chosen patients, both treatment modalities can acquire a satisfactory profile improvement.

## Figures and Tables

**Fig. (3):**
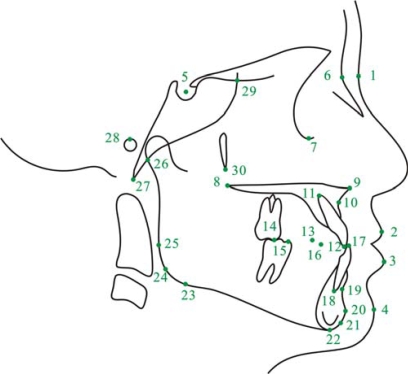
Landmarks used in this study: 1, soft-tissue nasion; 2, labrale superius; 3, labrale inferius; 4, soft-tissue pogonion; 5, sella; 6, nasion; 7, orbitale; 8, posterior nasal spine; 9, anterior nasal spine; 10, point A; 11, upper incisor apex; 12, incision superius; 13, upper first premolar tip; 14, upper molar mesial cusp tip; 15, lower molar mesial cusp tip; 16, lower first premolar tip, 17, incision inferius 18, lower incisor apex; 19, point B; 20, pogonion; 21, gnathion; 22, menton 23, lower gonion; 24, gonion; 25, posterior gonion; 26, arti-culare; 27, basion; 28, porion; 29, sphenoethmoidal point; 30, ptery-gomaxillare.

**Fig. (4):**
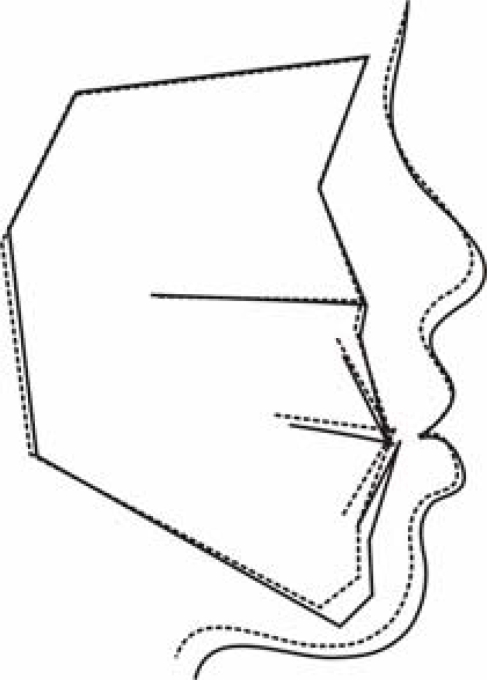
Superimposition of averaged pretreatment tracings of orthodontic and surgical groups along S-N at sella. Orthodontic group (dashed line); Surgical group (solid line).

**Fig. (5):**
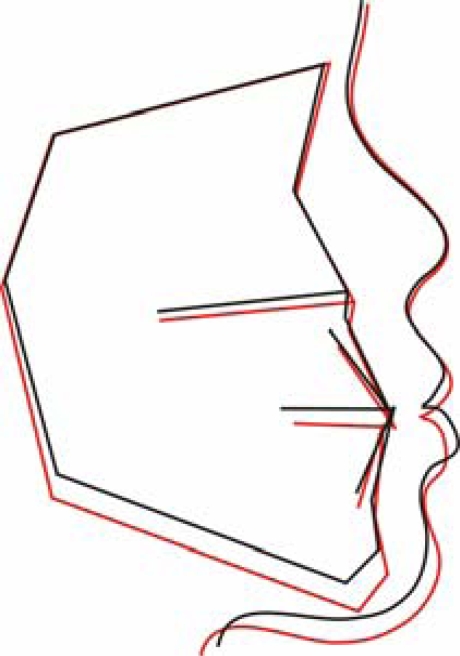
Superimposition of averaged pretreatment and posttreatment tracings within orthodontic group along S-N at sella. Pretreatment (black line); Posttreatment (red line).

**Fig. (7):**
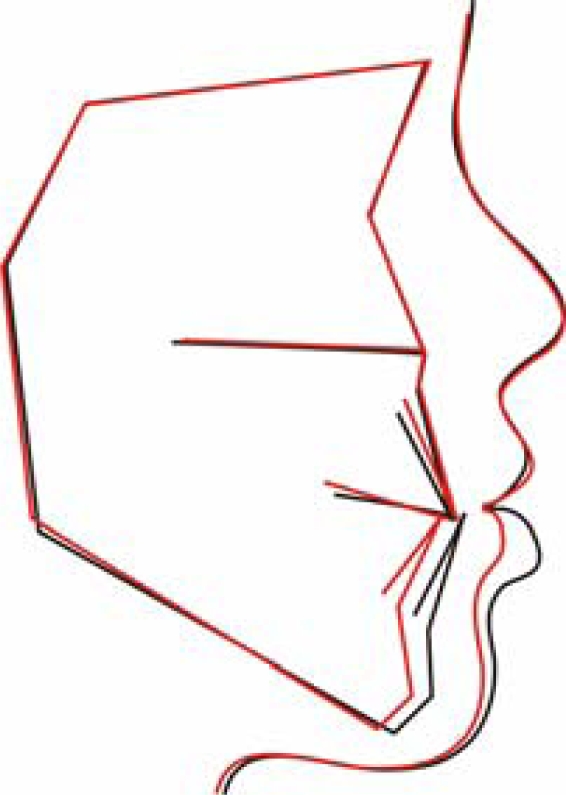
Superimposition of averaged pretreatment and posttreatment tracings within surgical group along S-N at sella. Pretreatment (black line); Posttreatment (red line).

**Fig. (8):**
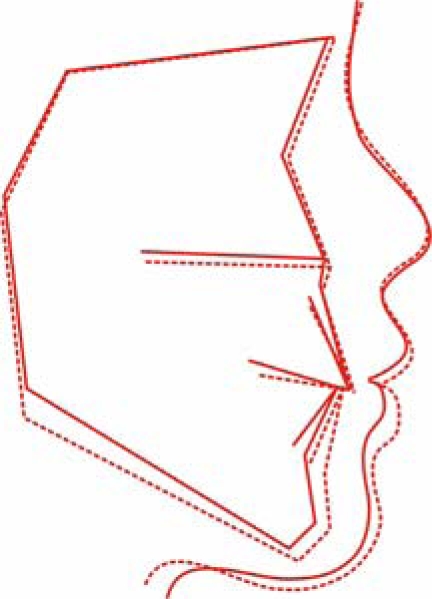
Superimposition of averaged posttreatment tracings of orthodontic and surgical groups along S-N at sella. Orthodontic group (dashed line); Surgical group (solid line)

**Table 1.** Details of Extraction Protocols in the Orthodontic Group

Extracted Teeth	Total (n=13)
14, 24, 34, 44	8
15, 25, 34, 44	2
13, 23, 34, 44	1
14, 25, 34, 44	1
34, 44	1

**Table 2.** Comparison of the Pretreatment Values for the between Orthodontic and Surgical Groups

	Pre-Treatment Orthodontic Group		Pre-Treatment Surgical Group		Mann-Whitney U Test
	Mean	SD	Mean	SD	Sig.
*Cranial base*					
PoOr-NBa(°)	27.45	1.82	28.86	3.91	NS
NSAr(°)	123.64	5.45	123.37	6.04	NS
BaSN(°)	131.47	4.39	131.49	5.00	NS
*Maxillary*					
SNA(°)	79.89	2.67	80.96	5.08	NS
PP-SN(°)	8.76	2.18	9.98	3.50	NS
*Mandibular*					
SNB(°)	81.35	2.81	83.08	6.60	NS
ML-SN(°)	33.84	5.23	35.65	6.32	NS
NPog-SN(°)	81.89	2.55	83.43	6.09	NS
Go-Me:S-N	111.38	8.22	118.99	9.10	*
*Maxillary/Mandibular*					
ANB(°)	-1.46	2.06	-2.12	2.51	NS
Wits(mm)	-8.46	2.73	-10.86	5.61	NS
PP-ML(°)	25.08	5.56	25.67	6.97	NS
M/M ratio	0.85	0.07	0.83	0.10	NS
NAPog(°)	-3.71	5.09	-3.61	7.07	NS
*Vertical*					
ArGoMe(°)	121.23	4.28	123.71	9.62	NS
Goupper(°)	45.65	3.43	45.23	4.41	NS
Golower(°)	75.58	4.77	78.49	7.01	NS
Facial Prop	55.43	2.71	56.28	2.49	NS
Y-axis(°)	61.43	4.08	60.06	3.44	NS
*Dentoaleolar*					
U1-SN(°)	111.76	6.02	108.74	11.07	NS
L1-ML(°)	93.74	7.30	86.91	10.97	NS
U1-L1(°)	120.65	7.89	128.71	10.95	*
*Soft tissure*					
Holdaway angle	14.57	4.07	10.14	4.26	*
Z angle	66.77	7.85	73.99	10.64	NS

NS, not significant; * P<0.05; ** P<0.01; *** P<0.001.

**Table 3.** Classification Results of Stepwise Discriminant Analysis

Original Group Membership	Predicted Group Membership	
	Orthodontic Group	Surgery Group
Orthodontic group	76.9% (n=10)	23.1% (n=3)
Surgery group	33.3% (n=4)	66.7% (n=8)

**Table 4.** Comparison of the Pretreatment and Posttreatment Values within the Orthodontic Group

	Pre-Treatment Orthodontic Group		Post-Treatment Orthodontic Group		Wilcoxon Signed Rank Test
	Mean	SD	Mean	SD	Sig.
*Cranial base*					
PoOr-NBa(°)	27.45	1.82	28.17	2.07	*
NSAr(°)	123.64	5.45	123.67	5.61	NS
BaSN(°)	131.47	4.39	132.41	5.20	NS
*Maxillary*					
SNA(°)	79.89	2.67	79.11	3.49	NS
PP-SN(°)	8.76	2.18	9.00	2.97	NS
*Mandibular*					
SNB(°)	81.35	2.81	80.79	2.84	NS
ML-SN(°)	33.84	5.23	33.65	6.16	NS
NPog-SN(°)	81.89	2.55	81.82	3.03	NS
Go-Me:S-N	111.38	8.22	114.43	7.07	**
*Maxillary/Mandibular*					
ANB(°)	-1.46	2.06	-1.68	1.54	NS
Wits(mm)	-8.46	2.73	-7.23	3.22	NS
PP-ML(°)	25.08	5.56	24.65	6.31	NS
M/M ratio	0.85	0.07	0.85	0.05	NS
NAPog(°)	-3.71	5.09	-5.33	5.04	NS
*Vertical*					
ArGoMe(°)	121.23	4.28	119.44	3.87	**
Goupper(°)	45.65	3.43	43.69	3.71	**
Golower(°)	75.58	4.77	75.74	5.16	NS
Facial Prop	55.43	2.71	56.13	2.62	*
Y-axis(°)	61.43	4.08	61.03	3.91	NS
*Dentoalveolar*					
U1-SN(°)	111.76	6.02	110.21	4.88	NS
L1-ML(°)	93.74	7.30	86.65	6.59	**
U1-L1(°)	120.65	7.89	129.48	5.61	**
*Soft tissure*					
Holdaway angle	14.57	4.07	13.46	4.87	NS
Z angle	66.77	7.85	74.94	9.29	**

NS, not significant; * P<0.05; ** P<0.01; *** P<0.001.

**Table 5.** The Values of the Retraction of the Lower Incisors in the Orthodontic Group (n=13)

	Mean	SD
Incisal tip (mm)	4.88	2.77
Incisal apex (mm)	1.92	1.78

**Table 6.** Comparison of the Pretreatment and Posttreatment Values within the Surgical Group

	Pre-Treatment Surgical Group		Post-Treatment Surgical Group		Wilcoxon Signed Rank Test
	Mean	SD	Mean	SD	Sig.
*Cranial base*					
PoOr-NBa(°)	28.86	3.91	28.49	3.40	NS
NSAr(°)	123.37	6.04	123.72	5.00	NS
BaSN(°)	131.49	5.00	132.53	5.84	NS
*Maxillary*					
SNA(°)	80.96	5.08	80.91	4.63	NS
PP-SN(°)	9.98	3.50	9.53	3.68	NS
*Mandibular*					
SNB(°)	83.08	6.60	79.62	5.12	**
ML-SN(°)	35.65	6.32	37.01	5.31	NS
NPog-SN(°)	83.43	6.09	81.38	4.86	*
Go-Me:S-N	118.99	9.10	117.02	7.71	NS
*Maxillary/Mandibular*					
ANB(°)	-2.12	2.51	1.30	2.36	**
Wits(mm)	-10.86	5.61	-4.85	3.60	**
PP-ML(°)	25.67	6.97	27.48	5.94	NS
M/M ratio	0.83	0.10	0.93	0.12	**
NAPog(°)	-3.61	7.07	-0.97	4.49	NS
*Vertical*					
ArGoMe(°)	123.71	9.62	124.19	9.64	NS
Goupper(°)	45.23	4.41	45.56	4.92	NS
Golower(°)	78.49	7.01	78.64	6.34	NS
Facial Prop	56.28	2.49	56.58	2.87	NS
Y-axis(°)	60.06	3.44	61.05	4.04	NS
*Dentoaleolar*					
U1-SN(°)	108.74	11.07	107.28	8.23	NS
L1-ML(°)	86.91	10.97	94.02	7.96	**
U1-L1(°)	128.71	10.95	121.70	7.17	NS
*Soft tissure*					
Holdaway angle	10.14	4.26	14.72	2.90	**
Z angle	73.99	10.64	75.71	4.85	NS

NS, not significant; * P<0.05; ** P< 0.01; *** P<0.001.

**Table 7.** Comparison of the Posttreatment Values between the Orthodontic and Surgical Groups

	Post-Treatment Orthodontic Group		Post-Treatment Surgical Group		Mann-Whitney U Test
	Mean	SD	Mean	SD	Sig.
*Cranial base*					
PoOr-NBa(°)	125.90	129.50	28.49	3.40	NS
NSAr(°)	48.63	42.34	123.72	5.00	NS
BaSN(°)	132.41	5.20	132.53	5.84	NS
*Maxillary*					
SNA(°)	79.11	3.49	80.91	4.63	NS
PP-SN(°)	9.00	2.97	9.53	3.68	NS
*Mandibular*					
SNB(°)	80.79	2.84	79.62	5.12	NS
ML-SN(°)	33.65	6.16	37.01	5.31	NS
NPog-SN(°)	81.82	3.03	81.38	4.86	NS
Go-Me:S-N	114.43	7.07	117.02	7.71	NS
*Maxillary/Mandibular*					
ANB(°)	-1.68	1.54	1.30	2.36	**
Wits(mm)	-7.23	3.22	-4.85	3.60	NS
PP-ML(°)	24.65	6.31	27.48	5.94	NS
M/M ratio	0.85	0.05	0.93	0.12	*
NAPog(°)	-5.33	5.04	-0.97	4.49	**
*Vertical*					
ArGoMe(°)	119.44	3.87	124.19	9.64	NS
Goupper(°)	43.69	3.71	45.56	4.92	NS
Golower(°)	75.74	5.16	78.64	6.34	NS
Facial Prop	56.13	2.62	56.58	2.87	NS
Y-axis(°)	61.03	3.91	61.05	4.04	NS
*Dentoaleolar*					
U1-SN(°)	110.21	4.88	107.28	8.23	NS
L1-ML(°)	86.65	6.59	94.02	7.96	*
U1-L1(°)	129.48	5.61	121.70	7.17	**
*Soft tissure*					
Holdaway angle	13.46	4.87	14.72	2.90	NS
Z angle	74.94	9.29	75.71	4.85	NS

NS, not significant; * P<0.05; ** P< 0.01; *** P<0.001.
